# Transposition of Teeth

**Published:** 1858-01

**Authors:** 


*Transposition of Teeth.*—Examples of the transposition of teeth, though somewhat rare, are nevertheless occasionally met with. Some years ago we gave a description of a case which we had seen, and about four weeks ago we saw another. The transposed teeth in the last case were the lower right lateral incisor and cuspidatus—the former occupying the place of the latter and the latter that of the former. The cuspid tooth in every other respect was regularly arranged, but the lateral incisor was partly turned upon its axis—the lingual surface of the crown being in contact with the first bicuspid and the labial surface in contact with the cuspidatus.

